# POPLITEAL ARTERY ARTERIOVENOUS FISTULA FOLLOWING KNEE ARTHROSCOPIC SURGERY

**DOI:** 10.51894/001c.123083

**Published:** 2024-08-30

**Authors:** Harminder Sandhu

**Affiliations:** 1 College of Osteopathic Medicine Michigan State University

## 77

### INTRODUCTION

Arthroscopic surgery, especially of the knee, is a relatively safe minimally invasive procedure with low rates of complications. Rarely this procedure can be associated with traumatic arteriovenous fistula (AVF) formation secondary to popliteal arterial injury. Here we present the case report of a 33-year-old female with symptomatic iatrogenic popliteal arteriovenous fistula formation following right knee arthroscopy.

### CASE DESCRIPTION

The case is a 33-year-old female with a 10-year history of left knee pain secondary to an unspecified knee injury. MRI revealed loose bodies within the knee joint and a medial meniscal tear. She underwent left knee arthroscopy with the removal of loose bodies, partial medial meniscectomy, medial femoral chondroplasty and debridement. Two weeks following arthroscopy, the patient developed new-onset left calf pain, edema, tenderness, and left lower extremity claudication with difficulty ambulating. Physical exam and imaging workup revealed a left popliteal arteriovenous fistula and associated venous pseudoaneurysm. The patient then underwent open surgical takedown of the arteriovenous fistula with patch angioplasty repair of the popliteal artery and primary repair of the popliteal vein and associated pseudoaneurysm. Postoperatively the patient progressed well with resolution of calf pain, edema and return of ambulation to her baseline.

### DISCUSSION/CONCLUSIONS

Traumatic AVF formation following iatrogenic vascular injury during knee arthroscopy is a rare phenomenon. These lower extremity AVFs can lead to devastating limb-threatening complications, especially with delayed diagnosis and treatment. Despite our patient having a good physical outcome following AVF repair, this complication could have led to permanent disability and limb loss if not identified and addressed in a timely fashion. Physicians should have a high index of suspicion for the possibility of vascular injury after knee arthroscopy, especially if surgery involves the knee’s posterior compartment. Preventative measures include avoiding unnecessary trauma, ensuring knee flexion, and avoiding excessive manipulation of the knee during surgery. This case adds to the limited literature on the topic of iatrogenic popliteal AVF formation as a potential complication of orthopedic surgery and discusses the prevention, diagnosis, and repair of vascular injury following knee arthroscopy.

